# Examining the relationship between depression and medication adherence among elderlies suffering from cardiovascular disease referring to the clinics affiliated with Kermanshah University of Medical Sciences: A cross‐sectional study

**DOI:** 10.1002/hsr2.1503

**Published:** 2023-08-17

**Authors:** Mina Brimavandi, Parvin Abbasi, Behnam Khaledi‐Paveh, Nader Salari

**Affiliations:** ^1^ Department of Nursing, Student Research Committee Kermanshah University of Medical Sciences Kermanshah Iran; ^2^ Department of Nursing, School of Nursing and Midwifery Kermanshah University of Medical Sciences Kermanshah Iran; ^3^ Department of Psychiatry Nursing, School of Nursing and Midwifery Kermanshah University of Medical Sciences Kermanshah Iran; ^4^ Department of Biostatics, School of Nursing and Midwifery Kermanshah University of Medical Sciences Kermanshah Iran

**Keywords:** cardiovascular, depression, medication adherence, older

## Abstract

**Background:**

In recent years, around 30% of all mortalities worldwide has been related to cardiovascular disease (CVD). The most important predictor solution of cardiovascular events is enhancing medication adherence. Meanwhile, the main reason behind development of physical disorders among the elderly is depression. In this regard, the present research was performed to determine the relationship between depression and medication adherence among the elderly suffering from CVD.

**Methods:**

A cross‐sectional study was done via an analytical approach on 188 elderlies fulfilling the inclusion criteria. The participants were chosen through convenience nonrandomized sampling from March to July 2022. The data were collected through demographic and healthcare information form plus Madanloo chronic disease medication adherence and geriatric depression scale. The analysis of the data was done by SPSS 26 plus Stata 14.2.

**Results:**

The mean depression score was 5.6(4.3) and the mean score of medication adherence in the elderlies was 168.03(23.85). The prevalence of depression was higher in women than in men (*p* = 0.015), and the elderlies differing from heart failure reported the minimum extent of medication adherence. The findings obtained from analysis of variance showed that age, level of education, and monthly level of income were among the effective demographic factors in the extent of depression and medication adherence (*p* < 0.05). Meanwhile, 26% of changes in medication adherence can be attributed to depression. Further, the results of the multiple linear regression model reported that depression, age, and polypharmacy are among the predictors of medication adherence.

**Discussion and Conclusion:**

A weak to moderate relationship existed between depression and medication adherence among these elderlies. Given the growing elderly population, the importance of depression, and lack of medication adherence in incidence of CVD events, it is recommended to train the healthcare team to monitor the elderly regarding depression symptoms.

## 1 INTRODUCTION

Aging is a period of life which begins from the age of 60 to 65 in Developed countries, and one of its consequences is incidence of chronic diseases.^1^, ^2^ Aging itself does not cause disease, rather through natural physiological changes of this period, it accelerates incidence of diseases.^1^, ^3^ Over recent decades, with an increase in the elderly population, the prevalence of chronic diseases has increased, where around 80% of elderlies suffer from at least one type of chronic disease, and 77% of elderlies endure two comorbidities.^4^, ^5^

Depression is the most common chronic psychiatric disorder in the elderly.^6^, ^7^ The prevalence of depression among elderly worldwide is 10%–15%.^2^, ^8^ In Iran, around 35%–45% of the elderlies have depression, with 30% of them not showing clear symptoms or undiagnosed.^2^, ^6^, ^9^ Depression affects the emotions, thoughts, and performance of the person, causing a wide range of physical and psychological problems in them.^10^ Meanwhile, cardiovascular disease (CVD), as a chronic physical illness, is the main cause of elderly mortality both globally and in Iran, and as such is the most important chronic disease among the elderlies.^5^, ^11^, ^12^, ^13^, ^14^ The chronic nature of diseases especially in the elderlies, by affecting the quality of life, the course of treatment, numbers of hospitalization, and eventually increasing the healthcare costs cause various consequences on their life.^15^, ^16^, ^17^ The elderlies suffering from CVD, due to awareness of the nature of CVD, the necessity of treatment follow‐up regularly in the long run, reduction of independence and compulsion in changing the daily and recreational activities due to reduction in physical abilities, have to endure great psychological burden.^18^, ^19^ As such, they are more at risk of developing depression. Global statistics have predicted that by 2030, depression and CVD would be known as two important causes of disability.^20^

Medication adherence refers to extent of person's adherence to health‐related behaviors including taking medications, following the proper diet, and having a healthy lifestyle according to the healthcare team recommendations.^21^ Based on the results of different studies, medication adherence among the elderlies suffering from CVD is 50% lower compared with young individuals.^22^, ^23^, ^24^ Thus, medication adherence is an important variable for determining the extent and severity of disease complications in the elderlies.^25^

Based on the results of a number of studies, depression and self‐efficacy among the older patients with hypertension (HTN), as a type of CVD, is an important predictor of medication adherence.^26^ In this regard, early diagnosis and treatment of depression can be one of the most important factors behind treatment and control of HTN in elderlies.^27^ Meanwhile, passage of a long time past the disease diagnosis can cause increased extent of medication adherence among elderlies suffering from CVD and depression simultaneously since the elderly has become accustomed to adhering to health‐related recommendations.^28^

Considering the importance of two variables of depression and medication adherence on each other, lack of studies in this topic in Iran, the existence. Significantly different results in this regard,^26^, ^27^, ^28^, ^29^ and reduction of psychological health level of the elderlies through the COVID‐19 pandemic, this study aimed to explore the effect of depression on medication adherence among elderlies suffering from CVD.

## 2 MATERIALS AND METHODS

### 2.1 Study design

This study is a descriptive analytic cross‐sectional study undertaken from March to July 2022.

### 2.2 Participants

The research population consisted of all older patients with CVD and referred to the clinics affiliated with Kermanshah University of Medical Sciences (Shahid Fatahi, Bustan, and Mahdieh). The subjects were included through convenience nonrandomized sampling. The inclusion criteria were: (1) age 60 years and above, (2) having at least one type of CVD, (3) no history of having psychiatric or psychological disorders, (4) not taking psychiatric drugs at the time of entrance to the study, (5) ability to answer the questions, (6) informed consent for participation in the research. Incomplete responses to at least one of the items caused the participant to be exclude from the study.

### 2.3 Study instruments

In this study employed two questionnaires, Geriatric Depression Scale (GDS), and Madanloo chronic disease medication adherence questionnaire. Also, the demographic information form is used.

The demographic information form is a researcher‐made form which was developed by researchers based on previous studies (it included 22 items about personal information such as age, gender, education, marital status, place of residence, type of residence, occupational status, monthly level of income, current history of smoking, physical activity and insurance status, clinical information including type of CVD, duration past the disease diagnosis, number of drugs taken a day, familial history of CVD, having another comorbidity, history of stroke, history of hospitalization in the past 1 year, history of occurrence of drug side effects, health status, need to continuous care and presence at clinic).

The elderly depression scale was developed in 1986 by Yasavij et al.^30^ The reliability and validity of this instrument have been confirmed in various studies. In Iran, in the study by Malakuti et al., the validity of this instrument has been examined and confirmed via criterion validity and factor analysis, and its reliability through Cronbach‐*α* coefficient (*α* = 0.9).^31^ This questionnaire has 15 items, composed based on binary scoring scale (1: yes, 0: no). Items 1, 5, 7, 11, 13, and 15 have been reverse scored (1: no, 0: yes). This questionnaire is the short form of the 30‐item questionnaire. The individuals, based on their acquired scores, would fall into one of the four following categories: no depression (0–4), mild depression (5–8), moderate depression (9–11), and severe depression (12–15). In this study, GDS questionnaire was also examined in terms of internal consistency across the target population, and its Cronbach‐*α* coefficient was 0.88.

The chronic disease medication adherence questionnaire was designed in 2013 by Madanloo et al. Its validity and reliability have been confirmed in various studies; in the study by Seyed Fatemi et al., the validity of this instrument has been confirmed based on quantitative and qualitative face validity as well as content and construct validity, while its reliability has been verified via test–retest method based on Cronbach‐*α* internal consistency coefficient (0.92).^32^ This questionnaire has 40 items and 7 subscale of persistence in treatment (items 1–9), willingness to participate in the treatment (items 10–16), adaptability (items 17–23), incorporating treatment into life (items 24–28), medication adherence (items 29–32), commitment to treatment (items 33–37), and discretion in treatment implementation (items 38–40). The scoring scale of this questionnaire is of 6‐point Likert scale type, including absolutely, very much, much, little, very little, and not at all, scored from 0 to 5, respectively. Items 25, 26, 33, 34, 35, 37, 38, 39, and 40 have been reverse scored. Based on their acquired scores, the subjects would fall into one of the four following categories: poor medication adherence (0%–25%), moderate medication adherence (26%–49%), good medication adherence (50%–75%), and medication adherence (76%–100%). In this study, again the Madanloo questionnaire was examined in terms of internal consistency, and the Cronbach *α* coefficient was 0.91.

### 2.4 Ethical considerations

The ethics committee of KUMS confirmed the study with the ethics code of IR.KUMS.REC.1400.607. The objectives of this study were explained to all subjects, and the confidentiality of their characteristics, as well as responses, were assured. Informed consent was also taken from all participants.

### 2.5 Data collection

First, permission for the study was taken from Kermanshah University of Medical Sciences. The researcher introduced herself to the manager of each clinic affiliated with KUMS. After explaining the research objectives and obtaining informed consent from participants, collecting the data from the elderly referring to the mentioned clinics and meeting the inclusion criteria is started. This stage is performed in waiting rooms of these clinics. Also, it lasted for 4 months.

### 2.6 Statistical analysis

The data were analyzed by SPSS 26 and Stata 14.2. The demographic characteristics, healthcare, depression, and various dimensions of medication adherence were analyzed by descriptive statistics. Kolmogorov–Smirnov test, skewness and kurtosis values were used for examining normality of the quantitative variables. The difference between the mean scores of depression and medication adherence in the groups of each demographic and clinical variable was analyzed by Mann–Whitney *U* and Kruskal–Wallis nonparametric tests along with *t* test and one‐way analysis of variance parametric tests. For analytical examination of the relationship between depression and medication adherence as well as its subgroups, Pearson's correlation coefficient was used. The predictors of various dimensions of medication adherence and depression were analyzed by multiple linear regression model. The level of significance for each statistical test was considered 0.05.

## 3 RESULTS

The data related to demographic and clinical variables among the 188 elderlies participating in this study are shown in Table 1. The age of the researched subjects was 60–89 years (66.61 ± 6.56), around half of whom having 60–64 years of age. Also, 61.2% of them were female, 76.6% were married, and 42.6% were illiterate. Further, 83.5% lived in cities, and 87.8% lived in their private houses. In addition, 11.2% were employed, and 56.9% had an income below 3 million Tomans per month. From among the researched elderlies, 38.3% had acute coronary syndrome, 28.2% HTN, and 30.0% had both of them. Further, more than 10 years had been passed from diagnosis of the disease of 22.9% of these elderlies. Additionally, 53.7% took fewer than five and 9.0% consumed more than 10 medications per day. The history of incidence of CVD in the first‐degree relatives was reported by 66.0% of the participants. Also, 69.7% suffered from other comorbidities. Nevertheless, 16.0% of these patients had no insurance coverage. Considering the variable of history of hospitalization over the previous year, 55.9% of them reported it as positive. Also, 43.6% had experienced drug side effects. Further, 17.6% of them considered their health status as good, while 23.4% needed constant care. Nevertheless, 57.4% had no activity and only 36.2% of them noted irregular jogging. About 20% of the researched elderlies smoked either currently or used to do it in the past.

**Table 1:** Distribution of demographic and clinical characteristics (*n* = 188).

Characteristics	*n* (%)
Age	
60–64	90 (47.9)
65–69	49 (26.1)
70–74	23 (12.2)
75–79	11 (5.9)
≥80	15 (8.0)
Gender	
Female	115 (61.2)
Male	73 (38.8)
Education	
Illiterate	80 (42.6)
School level	84 (44.7)
University	24 (12.8)
Marital status	
Married	144 (76.6)
Single, divorced or deceased spouse	44 (23.4)
Address	
City	157 (83.5)
Village	31 (16.5)
Home status	
Private or free	165 (87.8)
Rent	23 (12.2)
Work status	
At‐work	21 (11.2)
Retired	167 (88.8)
Income	
≤3millions	107 (56.9)
Between 3 and 5 millions	27 (14.4)
≥5 millions	54 (28.7)
Disease type	
Acute coronary syndrome (ACS)	72 (38.3)
Hypertension (HTN)	53 (28.2)
ACS + HTN	30 (16.0)
Heart failure (HF)	18 (9.6)
Other	15 (8.0)
Duration of physician's diagnosis	
≤1 year	41 (21.8)
Between 1 and 4 years	64 (34.0)
Between 5 and 9 years	40 (21.3)
≥10 years	43 (22.9)
Polypharmacy	
<5	101 (53.7)
Between 5 and 9	70 (37.2)
≥10	17 (9.0)
Family history of CVD	
Yes	124 (66.0)
No	64 (34.0)
Comorbidity	
Yes	131 (69.7)
No	57 (30.3)
Insurance	
Yes	158 (84.0)
No	30 (16.0)
Stroke history	
Yes	42.1
No	197.9
Hospitalization during the past year	
Yes	105 (55.9)
No	83 (44.1)
Drug side effects history	
Yes	82 (43.6)
No	106 (56.4)
Health status	
Poor	33 (17.6)
Moderate	95 (50.5)
Good	60 (31.9)
Constant care	
Yes	44 (23.4)
No	144 (76.6)
Accompanying status in clinic	
Alone	41 (21.8)
With family	147 (78.2)
Smoking status	
Yes	17 (9.0)
Never	148 (78.7)
Recent	23 (12.2)
Physical activity	
No activity	108 (57.4)
Regular	12 (6.4)
Walking	68 (36.2)

### 3.1 Prevalence of depression

**Figure 1:**
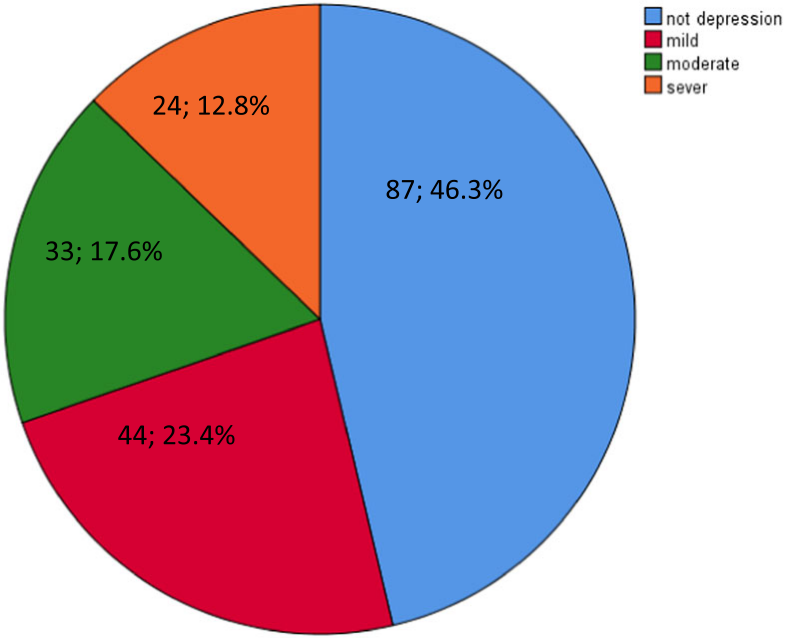
Frequency of depression status.

The prevalence of depression among the elderly suffering from CVD is reported in Figure 1. Accordingly, 46.3% of them had no signs of depression. Also, 23.4%, 17.6%, and 12.8% reported mild, moderate, and severe depression, respectively. Considering GDS, the mean depression among the elderlies was 5.6 ± 4.3.

### 3.2 Association between depression and demographic and clinical characteristics

The results of descriptive and inferential analysis related to the relationship between depression and demographic and clinical variables are listed in Table 2. Accordingly, 56.5% of the 70–74‐year‐old elderlies did report any sign of depression; in the 75–79‐year‐old age group, there was no elderly with severe depression. In addition, there was a significant correlation between depression and age, gender, education, marital status, monthly level of income, history of incidence of drug side effects, self‐report of health status, and physical activity (the results obtained from paired analysis of various groups of multichoice variables can be seen in Supporting Information: Table 1). Further, 70% of the elderlies who had an academic degree stated no symptoms of depression. In this regard, 65% of the elderlies who stated their health status as good and 75% of the elderlies who exercised regularly had no depression. Based on the *t* test results, depression was higher in women than in men. Also, less depression has been reported in married individuals.

**Table 2:** Comparison of demographic and clinical characteristics and depression statuses.

Characteristics	GDS depression status, *n*(%)				Test and significant
	No	Mild	Moderate	Severe	
Age					2.534
60–64	45(50.0)	20(22.2)	13(14.4)	12(13.3)	*p* value = 0.042*
65–69	22(44.9)	11(22.4)	9(18.4)	7(14.3)	
70–74	13(56.5)	7(30.4)	2(8.7)	1(4.3)	
75–79	4(36.4)	3(27.3)	4(36.4)	0(0)	
≥80	3(20.0)	3(20.0)	5(33.3)	4(26.7)	
Gender					2.45
Female	47(40.9)	25(21.7)	25(21.7)	18(15.7)	*p* value = 0.015*
Male	40(54.8)	19(26.0)	8(11.0)	6(8.2)	
Education					9.608
Illiterate	26(32.5)	20(25.0)	21(26.3)	13(16.3)	*p* value = 0.000*
School level	44(52.4)	20(23.8)	9(10.7)	11(13.1)	
University	17(70.8)	4(16.7)	3(12.5)	0(0)	
Marital status					2.425
Married	72(50.0)	36(25.0)	19(13.2)	17(11.8)	*p* value = 0.016*
Single, divorced or deceased spouse	15(34.1)	8(18.2)	14(31.8)	7(15.9)	
Address					0.939
City	72(45.9)	33(21.0)	30(19.1)	22(14.0)	*p* value = 0.352
Village	15(48.4)	11(35.5)	3(9.7)	2(6.5)	
Home status					1.732
Private or free	78(47.3)	40(24.2)	29(17.6)	18(10.9)	*p* value = 0.085
Rent	9(39.1)	4(17.4)	4(17.4)	6(26.1)	
Job status					0.996
At‐work	12(57.1)	4(19.0)	4(19.0)	1(4.8)	*p* value = 0.320
Retired	75(44.9)	40(24.0)	29(17.4)	23(13.8)	
Income					12.405
≤3 millions	42(39.3)	27(25.2)	23(21.5)	15(14.0)	*p* value = 0.000*
Between 3 and 5 millions	7(25.9)	9(33.3)	4(14.8)	7(25.9)	
≥5 millions	38(70.4)	8(14.8)	6(11.1)	2(3.7)	
Disease type					2.287
Acute coronary syndrome (ACS)	33(45.8)	17(23.6)	12(16.7)	10(13.9)	*p* value = 0.062
Hypertension (HTN)	30(56.6)	10(18.9)	7(13.2)	6(11.3)	
Heart failure (HF)	5(27.8)	4(22.2)	4(22.2)	5(27.8)	
ACS + HTN	15(50.0)	6(20.0)	7(23.3)	2(6.7)	
Other	4(26.7)	7(46.7)	3(20.0)	1(6.7)	
Duration of physician's diagnosis					0.700
≤1 year	18(43.9)	11(26.8)	7(17.1)	5(12.2)	*p* value = 0.553
Between 1 and 4 years	32(50.0)	17(26.6)	7(10.9)	8(12.5)	
Between 5 and 9 years	22(55.0)	5(12.5)	6(15.0)	7(17.5)	
≥10 years	15(34.9)	11(25.6)	13(30.2)	4(9.3)	
Polypharmacy					1.186
<5	46(45.5)	28(27.7)	12(11.9)	15(14.9)	*p* value = 0.308
Between 5 and 9	35(50.0)	14(20.0)	14(20.02)	7(10.00	
≥10	6(35.3)	2(11.8)	7(41.2)	2(11.8)	
Family history of CVD					0.818
Yes	61(49.2)	26(21.0)	20(16.1)	17(13.7)	*p* value = 0.415
No	26(40.6)	18(28.1)	13(20.3)	7(10.9)	
Comorbidity					0.219
Yes	61(46.6)	28(21.4)	26(19.8)	16(12.2)	*p* value = 0.827
No	26(45.6)	16(28.1)	7(12.3)	8(14.0)	
Insurance					1.539
Yes	76(48.1)	36(22.8)	27(17.1)	19(12.0)	*p* value = 0.125
No	11(36.7)	8(26.7)	6(20.0)	5(16.7)	
Stroke history					0.514
Yes	2(50.0)	1(25.0)	1(25.0)	0(0)	*p* value = 0.608
No	85(46.2)	43(23.4)	32(17.4)	24(13.0)	
Hospitalization during the past year					1.350
Yes	47(44.8)	21(20.0)	23(21.9)	14(13.3)	*p* value = 0.179
No	40(48.2)	23(27.7)	10(12.0)	10(12.0)	
Drug side effect's history					2.934
Yes	32(39.0)	16(19.5)	21(25.6)	13(15.9)	*p* value = 0.004*
No	55(51.9)	28(26.4)	12(11.3)	11(10.4)	
Health status					11.583
Bad	9(27.3)	7(21.2)	7(21.2)	10(30.3)	*p* value = 0.000*
Moderate	39(41.1)	25(26.3)	20(21.1)	11(11.6)	
Good	39(65.0)	12(20.0)	6(10.0)	3(5.0)	
Constant care					1.194
Yes	18(40.9)	9(20.5)	10(22.7)	7(15.9)	*p* value = 0.234
No	69(47.90	35(24.3)	23(16.0)	17(11.8)	
Accompanying status in clinic					1.835
Alone	22(53.7)	11(26.8)	5(12.2)	3(7.3)	*p* value = 0.068
With family	65(44.2)	33(22.4)	28(19.0)	21(14.3)	
Smoking status					1.119
Yes	5(26.4)	6(35.3)	4(23.5)	2(11.8)	*p* value = 0.329
Never	69(46.6)	32(21.6)	28(18.9)	19(12.8)	
Recent	13(56.5)	6(26.1)	1(4.3)	3(13.0)	
Physical activity					13.420
No activity	35(32.4)	27(25.0)	28(25.9)	18(16.7)	*p* value = 0.000*
Regular	9(75.0)	3(25.0)	0(0)	0(0)	
Walking	43(63.2)	14(20.6)	5(7.4)	6(8.8)	

* *p* < 0.05.

### 3.3 Prevalence of medication adherence

**Figure 2:**
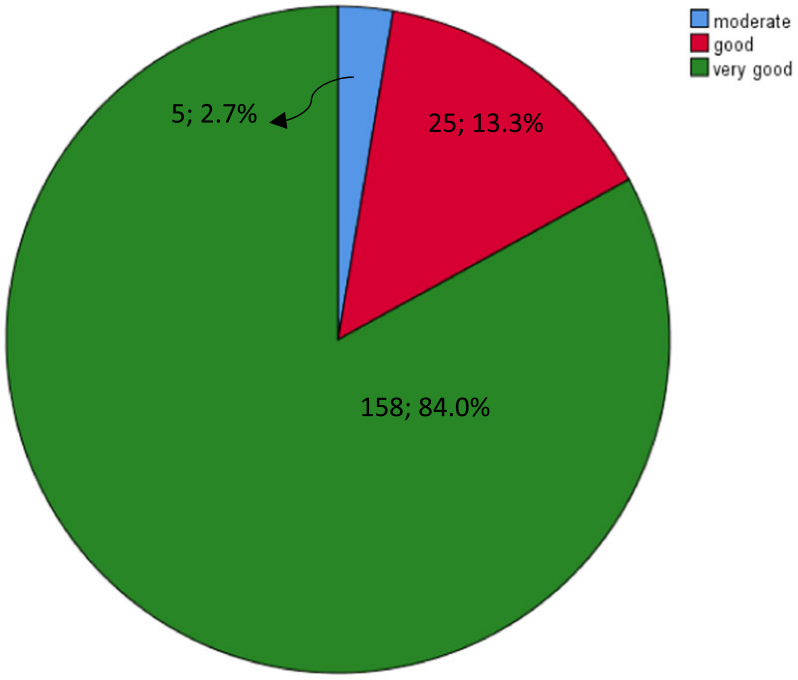
Frequency of medication adherence status.

Based on the descriptive analysis of the collected data and madanloo tool category, none of the elderlies reported a score lower than 50. Therefore, poor medication adherence is no detected in them. Meanwhile, 84% of them had very good medication adherence. Also, 13.3% and 2.7% of them reported good and average medication adherence, respectively. The mean score of medication adherence in these elderlies was 168.03 ± 23.85. The descriptive results related to this variable are shown in Figure 2.

### 3.4 Association between medication adherence and demographic and clinical characteristics

The results of descriptive and inferential analysis related to the extent of relationship between medication adherence and demographic and healthcare variables are reported in Table 3. There was a significant relationship between medication adherence and age, education, place of residence, occupational status, monthly income, type of disease, polypharmacy, presence at clinic, and previous history of cigarette smoking (paired analysis of different groups of multichoice variables is presented in Supporting Information: Table 2). As seen in Table 3, 93.3% of the elderlies aging 60–64 years old and 87% of women reported very good medication adherence. Also, all individuals who had regular physical activity had also very good medication adherence. Meanwhile, those suffering from both acute coronary syndrome and HTN, those with diagnosis past 10 years, and elderlies who consumed 10 drugs or more per day did not have poor or average medication adherence.

**Table 3:** Comparison of demographic and clinical characteristics and medication adherence status.

Characteristics	Medication adherence status, *n*(%)			Test and significant
	Moderate	Good	Very good	
Age				3.740
60–64	1(1.1)	5(5.6)	84(93.3)	*p* value = 0.006*
65–69	2(4.1)	12(24.5)	35(71.4)	
70–74	0(0)	4(17.4)	19(82.6)	
75–79	0(0)	2(18.2)	9(81.8)	
≥80	2(13.3)	4(26.7)	9(60.0)	
Gender				1.505
Female	2(1.70)	13(11.3)	100(87.0)	*p* value = 0.135
Male	3(4.1)	14(19.2)	56(76.7)	
Education				4.931
Illiterate	2(2.5)	12(15.0)	66(82.5)	*p* value = 0.008*
School level	1(1.2)	8(9.5)	75(89.3)	
University	2(8.3)	7(29.2)	15(62.5)	
Marital status				0.461
Married	3(2.1)	20(13.9)	121(84.0)	*p* value = 0.645
Single, divorced or deceased spouse	2(4.5)	7(15.9)	35(79.5)	
Address				2.295
City	5(3.2)	25(15.9)	127(80.9)	*p* value = 0.024*
Village	0(0)	2(6.5)	29(93.5)	
Home status				0.140
Private or free	5(3.0)	23(13.9)	137(83.0)	*p* value = 0.889
Rent	0(0)	4(17.4)	19(82.6)	
Job status				2.151
At‐work	2(9.5)	4(19.0)	15(71.4)	*p* value = 0.043*
Retired	3(1.8)	23(13.8)	141(84.4)	
Income (toman)				3.485
≤3 millions	2(1.9)	9(8.4)	96(89.7)	*p* value = 0.033*
Between 3 and 5 millions	0(0)	6(22.2)	21(77.8)	
≥5 millions	3(5.6)	12(22.2)	39(72.2)	
Disease type				4.240
Acute coronary syndrome (ACS)	1(1.4)	11(15.3)	60(83.3)	*p* value = 0.003*
Hypertension (HTN)	1(1.9)	7(13.2)	45(84.9)	
Heart failure (HF)	2(11.1)	3(16.7)	13(72.2)	
ACS + HTN	0(0)	2(6.7)	28(93.3)	
Other	1(6.7)	4(26.7)	10(66.7)	
Duration of physician's diagnosis				1.889
≤1 year	0(0)	5(12.2)	36(87.8)	*p* value = 0.133
Between 1 and 4 years	2(3.1)	11(17.2)	51(79.7)	
Between 5 and 9 years	3(7.5)	5(12.5)	32(80.0)	
≥10 years	0(0)	6(14.0)	37(86.0)	
Polypharmacy				3.931
<5	4(4.0)	18(17.8)	79(78.2)	*p* value = 0.021*
Between 5 and 9	1(1.4)	8(11.4)	61(87.1)	
≥10	0(0)	1(5.9)	16(94.1)	
Family history of CVD				0.168
Yes	2(1.6)	21(16.9)	101(81.5)	*p* value = 0.867
No	3(4.7)	6(9.4)	55(85.9)	
Comorbidity				0.778
Yes	4(3.1)	20(15.3)	107(81.7)	*p* value = 0.437
No	1(1.8)	7(12.3)	49(86.0)	
Insurance				0.328
Yes	4(2.5)	22(13.9)	132(83.5)	*p* value = 0.743
No	1(3.3)	5(16.7)	24(80.0)	
Stroke history				0.977
Yes	0(0)	2(50.0)	2(50.0)	*p* value = 0.330
No	5(2.7)	25(13.6)	154(83.7)	
Hospitalization during the past year				1.225
Yes	2(1.9)	13(12.4)	90(85.7)	*p* value = 0.222
No	3(3.6)	14(16.9)	66(79.5)	
Drug side effects history				1.614
Yes	2(2.4)	17(20.7)	63(76.8)	*p* value = 0.108
No	3(2.8)	10(9.4)	93(87.7)	
Health status				1.207
Poor	1(3.0)	5(15.2)	27(81.8)	*p* value = 0.301
Moderate	2(2.1)	17(17.9)	76(80.0)	
Good	2(3.3)	5(8.3)	53(88.3)	
Constant care				1.813
Yes	2(4.5)	9(20.5)	33(75.0)	*p* value = 0.075
No	3(2.1)	18(12.5)	123(85.4)	
Accompanying status in clinic				2.170
Alone	3(7.3)	11(26.8)	27(65.9)	*p* value = 0.095
With family	2(1.4)	16(10.9)	129(87.8)	
Smoking status				3.257
Yes	3(17.6)	3(17.6)	11(64.7)	*p* value = 0.041*
Never	2(1.4)	0(13.5)	126(85.1)	
Recent	0(0)	4(17.4)	19(82.6)	
Physical activity				2.908
No activity	4(3.7)	18(16.7)	86(79.6)	*p* value = 0.151
Regular	0(0)	0(0)	12(100)	
Walking	1(1.5)	9(13.2)	58(85.3)	

* *p* < 0.05.

### 3.5 Correlation coefficient between depression and medication adherence in elderlies with CVD

Based on statistical analysis of the obtained results, there was a significant and moderate relationship between depression and medication adherence. Also, the relationship between depression and all subgroups of medication adherence was inverse and significant, except for incorporation of treatment with life.

### 3.6 Multiple linear regression analysis predicting medication adherence

Among three predictor variables, all variables have affected medication adherence; every one unit increase in the extent of depression can reduce the medication adherence by 1.32 units. Also, every 1 year increase in age would lower medication adherence by 0.67. Considering the subgroups of polypharmacy variable, it can be stated that the elderlies who take 5–9 drugs and 10 drugs and more per day reported 8.62 and 14.51 units greater medication adherence compared with those who took fewer than 5 drugs (Table 4).

**Table 4:** Multiple linear regression analysis of the predictors of medication adherence.

Predictors		*B*	S.E	*β*	*p* Value
Depression		−1.32	0.40	−3.34	0.001
Age		−0.67	0.32	−2.09	0.038
Polypharmacy	Between 5 and 9	8.62	3.49	2.47	0.014
	More than 10	14.51	3.93	3.07	0.000
*R* ^2^		0.14			

## 4 DISCUSSION

The results of this study indicated that depression can lower the medication adherence in the elderlies suffering from CVD by up to 26%. The quality of life of the elderly especially their psychological health would be affected through the personal and social changes they face at the beginning and in the course of the aging period.^1^, ^33^ Meanwhile, depression is one of the most important consequences of CVD, which can increase mortality by up to 20% in these patients.^18^ In this study, 53.8% of the elderly suffered from varying levels of depression, and the mean depression score in the elderly felt into the mild category. In a study that had used 30‐item form of GDS, 57.1% of the elderly with HTN suffered from depression.^27^ The results of another study showed that 40.34% of the elderly suffering from coronary artery disease reported depression symptoms.^34^ Accordingly, it can be stated that the relationship between depression and CVD should receive the attention of the healthcare team, and the symptoms of depression in these elderlies should be monitored periodically.

In this study, the prevalence of depression in these older patients had a significant difference across the five categories based on age; so that, the elderly 80 years and above reported the highest mean score of depression. The results of a study indicated that age above 75 years can increase the prevalence of depression among the elderly suffering from HTN.^35^ However, another study stated that aging reduces the prevalence of depression among the elderly suffering from HTN by up to 23%.^36^ Based on the results of these studies, age is an important factor in the susceptibility of developing depression among the elderly suffering from CVD following the extent of caring for the disease.

Due to greater sensitivity to stressful events and higher prevalence of distress, women will develop depression more than men do.^37^ The results of this study also reported higher prevalence of depression in all of the categories, among women compared with men. It seems the way of life in Iranian women is different from men due to their cultural reasons. Accordingly, they take more care and responsibilities related to family members. A study performed on the elderly suffering from chronic diseases stated that depression is higher among women than in men.^38^ Other studies also suggested that depression is higher among women compared with men.^27^, ^35^

In this study, the prevalence of depression had a significant association with elderlies' education; the elderlies who were illiterate stated more symptoms of depression compared with their counterparts with primary, high school, and academic education. This was in line with several other studies.^27^, ^39^ It seems that higher levels of education are correlated with enhanced ability of establishing communication with others, especially in the form of mass and virtual media, as well as easier accessibility to information about health by elderlies, functioning as a preventive factor against depression.

Based on the results obtained from this study, there was a significant correlation between the prevalence of depression and marital status, whereby single, widowed, and divorced elderlies reported more depression compared with married individuals. Also, a study estimated that the odds of developing depression for single elderlies is 1.33 times greater than in married elderlies.^37^ Being married can be an effective factor against depression because of receiving emotional support by the spouse and increased life expectancy in elderly.

The monthly level of income is one of the factors that had a significant relationship with prevalence of depression among older patients with CVD. A study found that prevalence of depression is higher among the elderlies with income more than 5 million Tomans compared with those with less income. Based on the results of a study performed on the elderly with HTN, it can be stated that having higher income would reduce prevalence of depression.^27^ The results of other studies also confirmed this finding.^36^, ^40^ The extent of economic stability and its association with expectations in life can mediate the relationship between prevalence of depression and elderlies' income.

In the present study, there was a significant correlation between lack of depression and regular physical activity among the elderly. In a similar study on older patients with CVD, it has been reported that performing ideal physical activity can reduce the odds of developing depression by up to 35%.^40^ However, in another study examining elderlies with comorbidities, it was stated that excessive physical activity (more than 150 min per week) would elevate the odds of developing depression by 6.43 units.^41^ Researchers believe that having regular physical exercise programs is mostly a cultural belief rather than a health or rehabilitation issue. The commitment to physical activity in the context of clubs and under supervision of a trainer among Iranian elderlies is one of the important factors behind their low participation in physical activities.

The prevalence of depression in this study had a significant relationship with a positive history of experiencing drug side effects, which was in line with the results of another study. The results of this study show that the elderlies experiencing drug side effects showed increased depression development by 27%.^42^ Frequent elderly' visit by numerous specialists and the existence of a communication gap between different physicians would increase the probability of incidence of drug side effects through polypharmacy. Thus, the elderly become disappointed with the therapeutic effects of the drugs and are more susceptible to developing depression.

Depression has a relationship with good health self‐expression among the older patients with CVD. The elderlies who have chosen a healthy lifestyle have a better health status. Thus, their disease is under control and they are at less risk of developing depression. The results of a study indicated that there is an inverse relationship between better perceived health status and prevalence of depression.^27^ Another study also reported that good health status in elderly suffering from chronic diseases would lower prevalence of depression by 44%.^4^

No significant association was found between occupational status, comorbidity, as well as the constant need for care and depression. There was no association either between occupational status and depression among the elderly suffering from chronic heart failure, which was in line with the present study.^43^ Nevertheless, another study found employed elderlies have reported fewer symptoms of depression by 1.72 units compared with unemployed elderlies.^37^ The prevalence of depression in elderly suffering from HTN had no significant relationship with comorbidity^27^, ^44^; however, in other studies, there was a significant relationship between prevalence of depression and comorbidity.^36^, ^45^ Similar studies have shown a significant relationship between the constant need for care and prevalence of depression among the elderly.^4^, ^46^, ^47^, ^48^ It seems that personal and social differences of the elderly across different countries and even the cities of one country with varying cultures and attitudes would affect the extent of their distress considering their special conditions.

The mean score of medication adherence among the elderlies included in this study based on the Madanloo medication adherence questionnaire was into the very good category. One of the most important reasons of the elderlies' commitment to treatment was the good patient‐physician relationship. The results of a study that measured medication adherence with the same instrument suggest that those with CVD have good medication adherence.^49^ The results of a study reported average medication adherence (based on Murisky medication adherence instrument) among patients with HTN, and another study showed it as weak (based on PDC).^26^, ^50^ Cultural and racial differences in perception of disease and social support are important in the extent of medication adherence among elderlies across various regions.

The results of this study indicated that medication adherence has a significant relationship with the age of elderlies; medication adherence was higher by 51% among 60–69‐year‐old elderlies and with HTN, when compared with those 80 years of age and above.^51^ Meanwhile, the results of some studies state that there is no significant relationship between medication adherence and age of elderlies.^26^, ^27^, ^28^, ^34^, ^52^, ^53^ These studies have been done in countries, in which, unlike Iran, aging is not a newly emerging phenomenon and the elderly have adopted suitable self‐care behaviors.

There was a significant relationship between medication adherence and elderly' level of education. Elderlies who had academic degrees showed less medication adherence compared with other elderlies. Another study has shown that medication adherence among elderlies with academic degrees was 0.003 times lower compared with illiterate elderlies, which was statistically insignificant.^34^ However, there are also some studies reporting a direct relationship between medication adherence and higher levels of education.^36^, ^52^ Nevertheless, the results may imply that having the ability of reading and writing is one of the important factors in medication adherence among the elderly.

The results of this study also showed that there was a significant relationship between medication adherence and occupational status of elderlies. The elderly who were retired showed greater medication adherence compared with their employed counterparts. The extent of involvement of employed people with daily activities is greater compared with the retired ones, though probably this may vary considering the type of job among the elderlies of different countries. A study has indicated that medication adherence was 4% lower in elderlies who were never employed.^54^ Meanwhile, another study reported that medication adherence was 1.18 units lower in retired elderlies compared with their employed counterparts.^52^

In this regard, there was a significant relationship between medication adherence and the monthly level of income of elderlies. The elderly with a lower income reported better medication adherence. A study reported that higher income of elderlies would reduce the medication adherence slightly yet significantly.^34^ Meanwhile, the results of another study indicated that medication adherence was 13% greater in elderlies with a higher level of income compared with their low‐income counterparts.^55^ Around half of the employed elderlies in this study had income of greater than 5 million Tomans. Considering the interaction effect of the two variables of occupational status and the monthly level of income on each other, the findings of this research are justified.

A significant correlation was found between medication adherence and place of residence of elderlies; those living in villages showed greater medication adherence. A study on the elderlies suffering from HTN in Korea reported that the village‐dwelling elderlies had greater medication adherence compared with the elderlies of other cities (except for the capital city).^55^ Another study also confirms this finding and stated greater medication adherence among elderlies living in villages compared with city‐dwellers.^53^ It seems that the elderly' accessibility to economic as well as healthcare facilities is an influential factor affecting the extent of medication adherence and not merely the place of residence.

There was a significant difference between the extent of medication adherence in elderlies who had never smoked cigarettes and those who had been currently smoking or used to smoke. The results of another study also showed that cigarette smoking would reduce medication adherence by more than 200%, which is similar to the present study's findings.^29^ The results of some other studies also confirm this finding, albeit being insignificant.^53^, ^54^, ^56^, ^57^ Cigarette smoking is a voluntary activity and its harms are no secret to anyone. It is also one of the factors which is clearly in association with the extent of medication adherence.

The results of this study revealed that elderlies with HF had the minimum, while the elderlies with HTN and ACS concurrently had the greatest medication adherence, respectively. There was a significant relationship between the extent of medication adherence and the type of disease in the researched elderlies. The results of a study indicated that 80.2% of the elderly suffering from ACS had high medication adherence.^56^ The results of another study also confirmed this finding.^58^ Meanwhile, the results of another study showed that only 1.5% of patients with HF had high medication adherence.^59^ The nature of disease, being acute or chronic, heavily influences the extent elderly' care for controlling their disease.

The extent of medication adherence had a significant correlation with polypharmacy in the elderly. The results of a study showed that consuming fewer than four drugs per day by the elderly would enhance medication adherence by up to 51%.^51^ Nevertheless, some studies have reported no significant association between medication adherence and the number of drugs taken by the elderly per day.^27^, ^52^, ^53^ The results of different studies suggest that the most important reason behind lack of medication adherence among elderlies suffering from chronic diseases is polypharmacy.^60^, ^61^, ^62^, ^63^, ^64^, ^65^, ^66^ Nevertheless, the results of a systematic review have indicated that reduction of the number of drugs cannot be a major factor in enhancing medication adherence behaviors.^67^

Nevertheless, no significant association was found between medication adherence and gender, marital status, and the time past the disease diagnosis. Some studies have shown that there is no significant relationship between gender and medication adherence.^27^, ^57^, ^68^, ^69^, ^70^ A number of studies have reported no significant relationship between marital status and extent of medication adherence.^27^, ^28^, ^34^, ^58^, ^70^, ^71^ Finally, some studies have also stated that the duration past the disease diagnosis has no significant correlation with medication adherence.^34^, ^72^

The results of this study state that polypharmacy, taking 10 or more drugs, is a remarkable predictor for greater medication adherence. Since polypharmacy has been known as the most important cause of lack of medication adherence among the elderly suffering from chronic diseases.^61^ in recent studies, incidence of polypharmacy has been reported among different elderlies.^51^, ^67^, ^73^, ^74^ Meanwhile, considering depression as one of the predictor factors affecting lack of medication adherence, it seems that the healthcare team's commitment to monitory the elderlies with polypharmacy, such as awareness of the route of taking drugs, psychological health, caring for adherence to healthy diet and refraining from sedentary lifestyle, would be an effective step for enhancing medication adherence behaviors in these elderlies.

CVD patients are susceptible to developing depression because of the necessity of treatment follow‐up for a long time and lack of awareness of the chronic nature of CVD.^18^, ^22^, ^75^ Meanwhile, around 30% of the elderlies suffering from depression show no apparent symptoms.^2^ Further, comorbidity, polypharmacy, and lack of awareness about drugs cause poor medication adherence among the elderly.^12^, ^38^, ^76^, ^77^ In this study, it was also shown that medication adherence was significantly lower among the elderly suffering from CVD and depression compared with the elderly who showed no depression symptoms. A negative and mild‐to‐moderate relationship was also found between the mean score of depression and medication adherence in the researched elderlies. However, the results of some studies did not show a significant correlation between depression and medication adherence among the elderly suffering from HTN.^28^, ^55^ Other studies on elderlies with HTN, heart failure, or comorbidities confirm the results of this study.^16^, ^26^, ^34^, ^36^, ^38^

### 4.1 Limitations

Implementation of the study in only the clinics affiliated with the university and the elderlies referring to private clinics was one of the limitations. Also, the considerable crowd in these governmental clinics has been another limitation, which would complicate communication with the elderly. On the other hand, the number of items in the Madanloo medication adherence questionnaire was one of the other limitations of this study, which increased the time allocated to data collection for every participant.

## 5 CONCLUSION AND RECOMMENDATIONS

The findings of this study indicated that depression was higher in the elderly 80 years old and above, women, illiterate, single, widowed, and divorced subjects. Also, monthly income of more than 5 million Tomans, regular physical activity, history of incidence of drug side effects, and good health level had a significant relationship with no depression development. Further, 80‐year‐olds and above elderly, those with academic education, city dwellers, employed, and those with heart failure reported less medication adherence. Income less than 3 million Tomans per month, no history of cigarette smoking, and referral to the physician together with the family are among the factors reported among the elderly with greater medication adherence. Furthermore, there was a mild‐to‐moderate relationship between depression and lack of medication adherence. Aging, developing depression, and fewer number of drugs taken per day were identified as factors predicting greater extent of medication adherence. Since the complications resulting from depression and lack of medication adherence among the elderly suffering from CVD can cause irrecoverable consequences, attention to symptoms of depression, investigating the drug regimen, observing healthy lifestyle, and providing medication consultation by the healthcare team, especially nurses, physicians, and pharmacists seem useful. Furthermore, according to some unexpected and important results of this study that were mentioned above, presenting programs for raising awareness among the elderly about maintaining and managing their psychological and physical health is recommended.

## AUTHOR CONTRIBUTIONS

**Mina Brimavandi**: Resources; writing—original draft. **Parvin Abbasi**: Conceptualization; methodology; project administration; supervision; writing—review and editing. **Behnam Khaledi‐Paveh**: Conceptualization; writing—review and editing. **Nader Salari**: Data curation; formal analysis; methodology; validation.

## CONFLICT OF INTEREST STATEMENT

The authors declare no conflict of interest.

## ETHICS STATEMENT

The code of ethics has been approved by the Ethics Committee of Kermanshah University of Medical Sciences with code IR.KUMS.REC.1400.607.

## TRANSPARENCY STATEMENT

The lead author Parvin Abbasi affirms that this manuscript is an honest, accurate, and transparent account of the study being reported; that no important aspects of the study have been omitted; and that any discrepancies from the study as planned (and, if relevant, registered) have been explained.

## Supporting information

Supporting Information.

Supporting Information.

## Data Availability

The data set used and analyzed during the current study are available from the corresponding author on reasonable request (corresponding author: Dr. Parvin Abbasi, e‐mail: P_abasi2003@yahoo.com).
