# Supplementary material for: Examining the relationship between depression and medication adherence among elderlies suffering from cardiovascular disease referring to the clinics affiliated with Kermanshah University of Medical Sciences: A cross‐sectional study
Source: Health Sci Rep. 2023 Aug 17;6(8):e1503. doi: 10.1002/hsr2.1503 (PMC10435705; doi:10.1002/hsr2.1503)
Supplement: Supplementary file 1 — Supporting Information. [file HSR2-6-e1503-s002.docx]

Supplementary 1. Two-group analysis of depression scores and demographic and clinical characteristics using Bonferroni test

| variable | | | Mean difference | Std. Error | Sig. | 95% Confidence Interval | |
| --- | --- | --- | --- | --- | --- | --- | --- |
|  |  |  |  |  |  | Lower Bound | Upper Bound |
| Age | 70-74 | ≥ 80 | -4.14 | 1.41 | 0.036 | -8.12 | -0.15 |
| Education | Illiterate | Primary/high school | 1.93 | 0.64 | 0.009 | 0.37 | 3.48 |
|  |  | university | 3.87 | 0.96 | 0.000 | 1.55 | 6.18 |
| Income in month | ≤ 3 | ≥ 5 | 2.92 | 0.68 | 0.000 | 1.28 | 4.56 |
|  | 3 < income<5 |  | 4.06 | 0.96 | 0.000 | 1.74 | 6.37 |
| Physical activity | No activity | Regular | 4.21 | 1.23 | 0.002 | 1.24 | 7.19 |
|  |  | Walking | 2.81 | 0.63 | 0.000 | 1.29 | 4.32 |
| Health status | Good | Poor | -4.11 | 0.88 | 0.000 | -6.25 | -1.98 |
|  |  | Moderate | -2.19 | 0.67 | 0.004 | -3.82 | -0.57 |
