# Supplementary material for: Examining the relationship between depression and medication adherence among elderlies suffering from cardiovascular disease referring to the clinics affiliated with Kermanshah University of Medical Sciences: A cross‐sectional study
Source: Health Sci Rep. 2023 Aug 17;6(8):e1503. doi: 10.1002/hsr2.1503 (PMC10435705; doi:10.1002/hsr2.1503)
Supplement: Supplementary file 2 — Supporting Information. [file HSR2-6-e1503-s001.docx]

Supplementary 2. Two-group analysis of medication adherence scores and demographic and clinical characteristics using Bonferroni test

| variable | | | Mean difference | Std. Error | Sig. | 95% Confidence Interval | |
| --- | --- | --- | --- | --- | --- | --- | --- |
|  |  |  |  |  |  | Lower Bound | Upper Bound |
| Age | 60-64 | ≥ 80 | -21.68 | 6.47 | 0.010 | -40.07 | -3.28 |
|  | 70.74 |  | -23.63 | 7.70 | 0.025 | -45.52 | -1.74 |
| education | Primary/high school | university | 17.01 | 5.42 | 0.006 | 3.92 | 30.09 |
| Income in month | ≤ 3 | ≥ 5 | 9.66 | 3.94 | 0.045 | 1.51 | 19.16 |
| Smoking status | Never | yes | 15.37 | 6.04 | 0.035 | 0.76 | 19.16 |
| Disease type | HF | ACS | 19.36 | 6.09 | 0.017 | 2.07 | 36.66 |
|  |  | ACS+HTN | 26.23 | 6.89 | 0.002 | 6.67 | 45.80 |
